# An Entropy Generation Rate Model for Tropospheric Behavior That Includes Cloud Evolution

**DOI:** 10.3390/e25121625

**Published:** 2023-12-05

**Authors:** Jainagesh A. Sekhar

**Affiliations:** Department of Mechanical and Materials Engineering, University of Cincinnati, Cincinnati, OH 45221, USA; sekharja@uc.edu

**Keywords:** tropospheric entropy generation rate, global warming, climate, clouds, complex system behavior, intense weather, MEPR

## Abstract

A postulate that relates global warming to higher entropy generation rate demand in the tropospheric is offered and tested. This article introduces a low-complexity model to calculate the entropy generation rate required in the troposphere. The entropy generation rate per unit volume is noted to be proportional to the square of the Earth’s average surface temperature for a given positive rate of surface warming. The main postulate is that the troposphere responds with mechanisms to provide for the entropy generation rate that involves specific cloud morphologies and wind behavior. A diffuse-interface model is used to calculate the entropy generation rates of clouds. Clouds with limited vertical development, like the high-altitude cirrus or mid-altitude stratus clouds, are close-to-equilibrium clouds that do not generate much entropy but contribute to warming. Clouds like the cumulonimbus permit rapid vertical cloud development and can rapidly generate new entropy. Several extreme weather events that the Earth is experiencing are related to entropy-generating clouds that discharge a high rate of rain, hail, or transfer energy in the form of lightning. The water discharge from a cloud can cool the surface below the cloud but also add to the demand for a higher entropy generation rate in the cloud and troposphere. The model proposed predicts the atmospheric conditions required for bifurcations to severe-weather clouds. The calculated vertical velocity of thunderclouds associated with high entropy generation rates matches the recorded observations. The scale of instabilities for an evolving diffuse interface is related to the entropy generation rate per unit volume. Significant similarities exist between the morphologies and the entropy generation rate correlations in vertical cloud evolution and directionally solidified grainy microstructures. Such similarities are also explored to explore a generalized framework of pattern evolution and establish the relationships with the corresponding entropy generation rate. A complex system like the troposphere can invoke multiple phenomena that dominate at different spatial scales to meet the demand for an entropy generation rate. A few such possibilities are presented in the context of rapid and slow changes in weather patterns.

## 1. Introduction

### 1.1. The Earth’s Atmosphere

**Figure 1:**
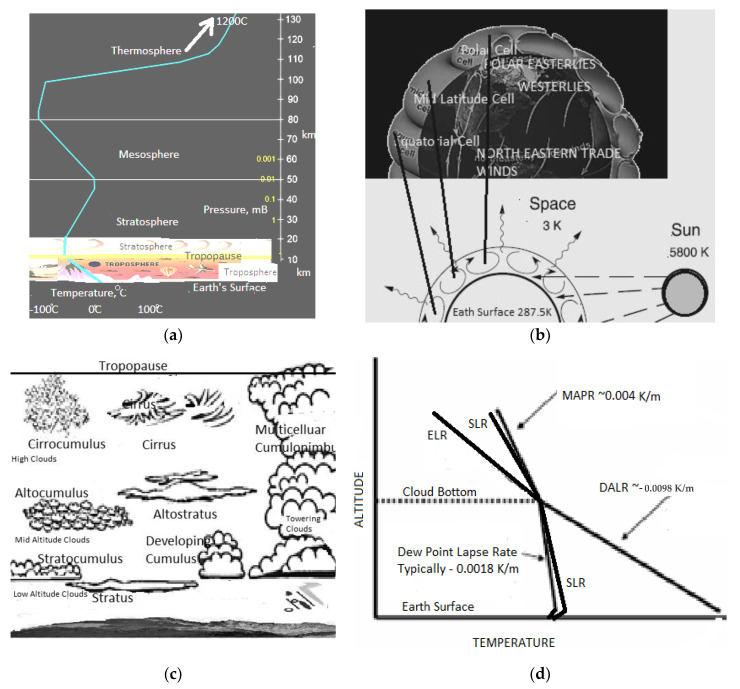
The figure illustrates all the major phenomena in the Earth’s atmosphere that will be important to the model and the discussions to follow. (**a**) The Earth’s atmosphere and temperature profiles across segments of the atmosphere. The troposphere is a small segment of the Earth’s atmosphere but contains almost all the weather and clouds; (**b**) the typical equatorial, tropical, and polar wind patterns in the northern hemisphere; (**c**) common types of clouds and their altitudes [1], (**d**) the typical temperature and various “lapse rates” in the troposphere—close to the Earth’s surface, lapse rate diurnal inversions are often noted, as shown in the schematic [1,2]. *Definitions:* A **cloud** is a mass of tiny water drops or ice crystals that float in the air above Earth. In this article, clouds are treated as diffuse interfaces. **Condensed water**: the liquid that is precipitated from a supersaturated H_2_O/air gas. **Cloud water content**: the cloud liquid water content is a measure of the total liquid water contained in a cloud in a vertical column of the atmosphere. It does not include solid water (snow, ice). **Precipitable water** is the amount of water potentially available in the atmosphere for precipitation, usually measured in a vertical column that extends from the Earth’s surface to the upper edge of the troposphere.

This article aims to provide a relationship between the Earth's changing weather and global warming by relating it to the demand in the tropospheric entropy generation rate. The climate we experience is related to weather events that involve clouds, precipitation, lightning, and wind in the troposphere. The troposphere contains about 80–85% of the atmosphere’s total mass and 99% of the water vapor, N_2_, O_2_, and aerosols. The average depth (or the tropopause level) of the troposphere is ~17 km (about 10.56 mi) in the middle latitudes, shallower at the cold poles ~7 km, and higher at the equator ~20 km. The troposphere is relatively shallow compared to the other layers of the atmosphere. However, the troposphere contains all atmospheric clouds except for a rare polar stratospheric cloud. The Earth’s surface temperature and surface texture strongly influence winds in the lower levels of the troposphere. Figure 1 illustrates the critical phenomena in the Earth’s atmosphere relevant to this article. Figure 1a shows the troposphere dimensions and temperature gradients in the atmospheric layers above the Earth’s surface. Figure 1b shows the typical wind patterns and radiation inlet and outlet into the troposphere. Figure 1c shows typical clouds observed in the troposphere. Figure 1d shows a typical temperature profile in the troposphere and provides a pictorial definition of some of the “lapse rate” temperature profiles discussed in the article. This article explores the relationship between the entropy generation rate in the troposphere and cloud morphology (patterns) for understanding weather. The caption of Figure 1 defines some of the parameters that are important for the model to follow.

The Earth’s average temperature now increases yearly (global warming) but shows diurnal and seasonal variations. The steady increase in the average from recent warming trends is dT_E_/dt~0.01 to 0.018 K/year or (3.17 to 5.7) × 10^−10^ K/s [3,4,5,6]. The absorption of infrared radiation by tropospheric gases is significant to the global energy balance. The increasing number of molecules in the Earth’s atmosphere that have three or more atoms, like water (H_2_O), methane (CH_4_), carbon dioxide (CO_2_), and nitrous oxides (NO_x_), absorb and transmit in the infrared spectrum, which leads to the Earth’s surface radiation being trapped, thus increasing the Earth’s surface and tropospheric warming. The average warming is accelerating for several reasons, including a lowering of the *albedo—*a measure of the proportion of incident radiation from outer space and the sun reflected by a surface. Since the greenhouse effect impacts clouds and ice melting, a change in albedo can contribute to overall warming temperatures. This article additionally considers emissivity changes in various cloud formations that impact the energy and entropy balance in the troposphere. 

Higher temperatures in the troposphere increase surface evaporation and, thus, influence the tropospheric water vapor content [3,4,5,6]. This is a consequence of the increased concentration of greenhouse gases in the troposphere (GHGs). The atmospheric moisture has increased since 1976. The troposphere's composition is ratio-wise uniform, except for water vapor. The water vapor proportion is usually highest near the surface and decreases with height [3,4,5,6]. For each 1 K warming, saturated air contains ~7 percent more water vapor on average [3,5,6]. The increase in the tropospheric water content has also led to more precipitation. When related to the warming Earth temperatures, the recorded precipitation increase is of the order of (1–3%/K) [3]. Changes in precipitation patterns are triggered by the increase in tropospheric moisture, which causes shifts in the tropospheric circulation, affecting the horizontal and vertical transport of water vapor [5,6]. 

The warming of the Earth’s surface temperature and tropospheric expands the troposphere. Recent evidence [4] suggests that the troposphere has undergone a significant rate of warming during the past century, equal to the warming of the Earth’s surface. The tropospheric temperature increase in the latter half of the 20th century is estimated to have risen at 0.01–0.018 K per year (like the surface temperature increase). The tropopause has expanded over the last forty years at approximately 5–10 m/year [4]. The study [4] also found that the troposphere expanded faster than in the 1900s after 2000–2020. This article attempts to provide a framework for the observed tropospheric phenomena and the tropospheric entropy demand rate. The article also explores the changes in cloud behavior, which influences precipitation.

### 1.2. Entropy Creation and Exchange

The behavior of open complex systems like the troposphere is difficult to model [7,8,9]. However, it is becoming recognized that the entropy generation rates, temperature distributions, chemical partitioning, fluid circulation patterns, and phase-boundary shapes are related [10], particularly when the system is at steady-state conditions [10,11,12,13,14,15,16,17,18,19,20,21,22] thereby allowing for new models to be explored such as the one presented in this article. The entropy generation rate has a relationship to pattern formations. Patterns and shapes have been studied with changing entropy generation rate principles for galaxy clusters, Hele-Shaw cells, natural convection, smokestacks, evolutionary biological systems, and directional freezing [10,13,14,15,16,17,18,19,20,21,22,23,24,25,26,27,28,29,30]. There is a scale at which free-energy dissipation and entropy generation are optimized by a system when it picks its dominant entropy generation mechanism [22]. Optimization of the entropy generation rate by changing the scale at which the new entropy is generated is often required by a complex system to alter the free-energy dissipation rate [11,12,13]. New patterns and phase boundaries emerge due to the changing demands inside an entropy-generating control volume [22]. Boundary defects with high-entropy regions are created to maintain a particular pattern of evolution [22]. Novel repetitive patterns are noted in open or cyclic systems where the energy changes its character, e.g., kinetic to potential energy or chemical to kinetic energy. For two-phase systems, a unique steady-state morphology is established for fixed forces and fluxes for an open system [13,17,18,19,22]. New morphological patterns can emerge from previous patterns when a change in the driving force occurs. The change in the type of patterns is enabled by amplifying or suppressing perturbations of a new type of order that maximizes and stabilizes the rate of entropy generation while attempting to approach steady-state conditions for an open thermodynamic system. Entropy-generating perturbations can occur at various scales, e.g., at the nanoscale by the flow of heat under a thermodynamic gradient (i.e., temperature gradient) or at the microscale with interface pattern evolution of a stable phase during a phase transformation. The system requirement that maximizes the entropy production rate sets the repetitive features of the new pattern. Because of the large possibilities of the force–flux relationship, the optimal patterns are not easily predictable, requiring experiments or simulations by analogs. 

The Earth maintains significant fluid circulation in the troposphere and the seas. Such circulation is enabled and maintained by rotation, friction, buoyancy, radiation, pressure gradients, tilt, and gravitational influences that impact the flux of matter and radiation which alter during diurnal, seasonal, and other changes. When the Earth’s average temperature is constant, an overall average entropy balance is maintained in the atmosphere, leading to somewhat predictable climate behavior. However, global warming distorts this previously established balance because it is difficult to establish steady-state conditions even of a cyclic nature. 

Entropy is transported by radiation from the sun to Earth across the tropopause into the troposphere. Entropy is also lost as radiation out of the troposphere and any jets of lightning that leave the troposphere. The Earth’s core also transports entropy to the troposphere, albeit in small amounts. If there is no unusual surface warming, there is no requirement for additional new entropy generation. When there is warming, the troposphere must generate new entropy for an entropy balance. It must also select and establish processes that can achieve the new rate of change requirements. *Here is where clouds most likely play a part.* Entropy generation in the troposphere can occur with heat-producing irreversible processes that lead to a loss of work potential. These include temperature, pressure, chemical concentration gradients, viscous dissipation, friction, turbulence cascades, and heat dissipation from the friction of two-phase movement enabled by gravity [31]. Non-equilibrium phase transformations with a diffuse interface, lighting jets escaping the troposphere, and changes in the type of cloud interfaces also produce new entropy. Calculations of the entropy generation in the troposphere have been made accurately [31,32,33,34]. However, the relationship to climate change has not yet been effectively established because of the complexity of understanding the weather patterns on a global scale from an entropy generation *rate* viewpoint. This article is a step in that direction, albeit with a low-complexity model.

Following the pioneering publications by Paltridge [32], Prigogine [11], and Zeigler [12], there have been competing arguments regarding entropy rate maximization or minimization for establishing stable pathways or morphological selections in a control volume. For pattern evolution during phase transformations, the entropy generation rate maximization postulate has predicted the correct diffusion coefficients [17]. Experimentally, it has been shown [17,22] that a near-reversible appearance of patterns is possible quickly with the cycling of process conditions, particularly when a steady state can be cyclically re-established [35,36,37,38,39,40,41,42]. There is less understanding of non-steady-state pattern evolution with unique metastable morphologies [37].

When thermal, biochemical, and chemical processes are stable with unchanging input and output rates in an open system, steady-state conditions are expected in the control volume where new entropy is produced [10,15,19,43]. The postulate of MEPR (maximum entropy production rate) compares the entropy generation rate for various patterns that can form at any scale during a process at a steady state. The principle of maximization of the entropy generation rate (MEPR) has led to accurate predictions of several parameters like the diffusion constant or geometric features like the pattern angle for bird flight and other experimentally verifiable results [18,22,23,31]. MEPR is equivalent to the maximum kinetic energy dissipation in a gravity-potential or the maximum heat transport in a temperature gradient.

### 1.3. Atmospheric Stability

The atmosphere’s stability describes its ability to resist or enhance vertical air movement. To maintain stability in the troposphere, the atmospheric temperature and pressure decrease with altitude, as required by the ideal gas law and gravity. Regardless, several atmospheric instabilities can manifest in the troposphere because of the Coriolis force, terrain, shifting wind masses, seasonal heating and cooling changes, the Earth’s tilt, and an imbalance in the carbon cycle caused by human activity causing greenhouse gas (GHG) emissions. This last part is expected to dominate the entropy generation demands in the troposphere; the main topic discussed in this article. There is now a growing recognition that atmospheric instabilities are increasing that correlate with the extreme temperatures, high winds, and rain, currently experienced on Earth. Extreme events are unusually severe weather or climate conditions that can cause devastating impacts on humans and asset values. Weather-related extreme events could be intense but short-lived, including lightning, heat waves, hail, damaging winds, heavy precipitation, flooding, tornadoes, tropical cyclones, and floods. The rising temperatures of the Earth are somehow causing extreme weather climate patterns. Extreme weather can be from the high temperatures directly associated with rising temperatures caused by heat entrapment (and a lack of daytime clouds) or associated with intense rain and hail associated with multicell (supercell) thunderstorms and high-velocity wind gusts (these require strong updrafts in clouds and high moisture in the updrafts).

Although not rigorously established in the existing literature, atmospheric instabilities caused by imbalances are rightfully thought to be related in some manner to increasing extreme weather events like intense heat, heavy rain, sleet, snow, and the increasing occurrence of atmospheric rivers, hurricanes, and tornados. This article aims to highlight the correlations between increasing surface temperature and atmospheric instabilities due to the increased demands of the entropy generation rate triggered by tropospheric warming. In this article, with the low-complexity model, a detailed parameterization of vertical convection [44], particularly regarding the interaction of horizontal and vertical movement of winds, is not included, except in the discussion section (Section 3) on tropospheric choices for the entropy-generating behavior and wind–cloud interactions.

### 1.4. Cloud Evolution and Intense Weather

Clouds represent a multiphase region (see definitions in Figure 1). Clouds form at altitudes where the dew-point temperature is higher than an air-parcel temperature, as shown in Figure 1c,d. In this article, clouds are studied as entropy-producing diffuse interfaces, in a manner previously studied for condensed-matter phase transformations [13,17,45].

Once formed by multiple nucleation events for precipitating condensed H_2_O, the clouds can stretch or connect horizontally or grow vertically. Horizontal clouds are not associated with external updrafts (see Table 1). Such clouds form at any altitude. When several nuclei are present, and a strong driving force for nucleation exists, the horizontal clouds form at low altitudes; otherwise, they form at mid to high altitudes (Figure 1b). Mid- and high-level thin clouds aid warming. Regardless of external updrafts or heterogeneous nuclei, the condensates in clouds, experience some undercooling before precipitation. The volume change and rapid heat release generated during the recalescence process cause local updrafts. When an updraft is possible, i.e., vertical movement is possible in the clouds, the clouds assume a cumulus character. The transition from flat to cumulus character is addressed in this article. It is known that horizontally layered stratus clouds are not *intense-weather-causing* phenomena unless instabilities manifest in the vertical direction. Cumulus clouds that only show slight vertical growth are associated with fair weather. When cumulus clouds begin to grow and resemble a head of cauliflower, they are called cumulus congestus, or swelling cumulus, which can further lead to towering cumulus. Vertical clouds are intense-weather-causing clouds. These clouds can develop into a giant cumulonimbus, i.e., thunderstorm clouds. The formation of rapidly growing vertical clouds that cause intense weather is favored by an unstable atmosphere, i.e., where strong updrafts are prevalent. Vertical clouds of the kind shown in Figure 1b are often the cause of intense weather with thunderstorms, hail, and lightning. Such clouds are studied in this article with a previously described entropy rate diffuse-interface model [17,45].

About two-thirds of Earth is typically covered by clouds. Clouds can impact the heating or cooling of the Earth’s surface depending on their altitude, thickness, water content, discharge, and coverage. Compared to a cloudless earth, the net effect of clouds is always to cool the Earth’s surface. Cooling by clouds is attributed to low-lying but vertically developing cumulus clouds. However, the presence of clouds can also heat the Earth by reflecting the radiation emitted by the Earth, an effect mainly attributed to high-altitude cirrus clouds [46,47]. The coverage of clouds changes with the average troposphere temperature [48]. Any changes in cloud patterns are thus expected to alter the troposphere’s radiative energy balance. In addition, the cloud-pattern changes may alter water exchanges (precipitation) that determine the weather. Clouds within a mile of the Earth’s surface cool the Earth’s surface. In high-altitude clouds, the warming effect usually outweighs the cooling, whereas the opposite is true for lower-altitude clouds [6,48,49,50]. At a low altitude, thick clouds reflect the sun’s heat (low-wavelength radiation). High, thin clouds trap some of the sun’s heat, thus warming the Earth’s surface. Observations have shown, however, that warmer temperatures seem to create less dense and low-level clouds [47]. Water or ice particles in clouds (depending on the type of cloud) can reflect between 30 and 60 percent of the sunlight that strikes them. The impact of the surface temperature change is particularly significant in the cloud coverage in the polar regions [46,47]. 

The condensation of water vapor in the atmosphere yields many small H_2_O particles with a post-nucleation droplet size of about 1 micrometer (micron). These tiny cloud droplets tend to be carried along with the vertically moving air because the flow required for suspension of even a 30-micron droplet is only 0.02 m/s—far lesser than the typical cloud updraft velocity of about ten m/s. The total water mixing ratio and entropy are conserved by parcels that have nucleated but are still in an updraft. The initially tiny starter cloud water droplets can coalesce into larger sizes greater than ~1 mm (about 0.04 in), which makes them challenging to suspend with the updraft velocity. Updrafts cannot carry larger droplets, e.g., a ~3 mm (about 0.12 in) water-particle diameter; the updraft must exceed 20 m/s to keep it suspended. Consequently, the large particles fall because of gravity. Maritime clouds have larger water-droplet sizes compared with land (continental) clouds. The droplet distribution typically varies between a few and 50 microns in clouds. As the water droplets and ice particles rise and coalesce, the droplets and ice crystals become larger and can no longer be supported by the cloud updraft. These large water droplets or hail fall to the ground, often with rapid downdrafts.

Thunderstorm-causing clouds reach a mature stage when updrafts, downdrafts, and circulation are simultaneously noted in the cloud with ensuing high-velocity winds, heavy rain/hail, thunder, lightning, and tornado developments. In the final stage of their lives, towering clouds mitigate their severe weather interaction with the Earth and dissipate quickly, albeit with strong winds and some residual lightning. Typically, the formation of a thundercloud starts with a 3 km broad cloud base, increasing to about ~7–10 km at maturity, which finally peters down to a few km broad cloud base. Tornadoes can develop from the interaction of updrafts and horizontal winds in thunderstorm clouds. The model presented in this article addresses rapid cloud evolution and dissipation and relates it to the far slower but more permanent global shifts in average Earth surface temperature.

Some clouds, like the cumulonimbus, are associated with lightning that strikes Earth, and also with charge-stream jets from the troposphere to the stratosphere. Lightning is generally produced during a developed stage of a thunderstorm-producing cloud with simultaneous updrafts and downdrafts in multicellular clouds. Such movements aid the separation of positive and negative charges that cause arc-like discharges (lightning). Lightning strikes have also increased over time, i.e., a higher frequency of lightning strikes has been recorded as the atmosphere warms. Climate models indicate that such strikes could increase from the current three strikes globally to four strikes per second with global warming [51]. There is a loss of entropy from the troposphere (the control volume in the model to follow) with thunderbolts and jets that escape the troposphere region. This is a mechanism for more entropy generation demand in the troposphere as well as an entropy generation mechanism within clouds. Jets and gigantic jets are upward electrical discharges from thundercloud-tops. They originate inside thunderstorms and escape from thundercloud tops, reaching 40–50 km and 70–90 km altitude, i.e., they cross the tropopause boundary and expel entropy from the tropopause. The upward currents from individual thunderstorms that do not return to Earth flow to the ionosphere, where they combine to establish the ionospheric potential.

The mass of water vapor in the atmosphere is only about 0.001 percent by mass (see Table 1 for different clouds). It is highest in the tropics and decreases toward the poles. About ~425,000 Km^3^ of water evaporates from the land and ocean surface annually, remaining for about ten days in the atmosphere before falling back to the surface as rain or snow/ice. The estimates are that 86% of global evaporation and 78% of global precipitation occur over the oceans; thus, a net water transfer occurs over land regions (the water is returned by riverways to the oceans). However, climate change is likely causing parts of the water cycle to speed up and change the return pathways as warming global temperatures increase the evaporation rate worldwide. On average, more evaporation causes more precipitation. Climate change affects the world’s water in complex ways, from unpredictable rainfall patterns to shrinking ice formations, rising sea levels, severe floods, and droughts. 

### 1.5. Lapse Rates

Various “lapse rate” nomenclature (see Figure 1) is used in climate models and in popular discussions of atmospheric sciences to explain atmospheric instabilities. Although called a rate, the lapse rate nomenclature refers to the temperature gradient. An atmospheric lapse rate is the rate of change in temperature observed while moving upwards through the Earth’s atmosphere. A few of the important lapse rates applicable to this article are discussed below. The thermodynamically established dry adiabatic lapse rate (DALR) equals (−g/*C_p_*), where g is the gravitational constant and *C_p_* is the specific heat_._ This is the lapse rate of unsaturated air and is roughly linear ~−9.76 K/km with altitude. When the temperature falls below the dew point, subject to overcoming nucleation difficulties, cloud formation is observed in the troposphere. Saturated air is at the dew-point temperature (a condition that depends on the water content and pressure). The local moist adiabatic lapse rate (MALR) is the lapse rate of saturated air with a condensed phase. The saturation and lower-than-saturation lapse rate (SLR) and the MAPR are seemingly similar for saturated and near-saturated conditions, but the SLR can extend below the cloud base. The dew point also has a lapse rate. It tracks the density profile of the air masses and is typically about ~−1.8 K/km. However, closer to the Earth’s surface, the dew point may also show a positive lapse rate. Some typical lapse rate illustrations are shown in Figure 1c.

The comparison of the lapse rate of a rising air parcel to the actual temperature conditions determines the condensation and vertical velocity of a parcel of air. This vertical velocity is the key to the development of cumulonimbus clouds that are associated with severe weather. The moist adiabatic lapse rate (MALR) and (SLR) (the near saturation lapse rate) typically vary between −4 and −9 K/km. An “average” value of about −4.5 K/km is reasonable to consider. The environmental lapse rate (ELR) is the actual lapse rate. Suppose the ELR-determined temperature at a given altitude is higher than the MALR or the dew-point temperature; the air will be stable, whether unsaturated or saturated. Suppose the ELR is more negative (steeper) than the dry adiabatic lapse rate; the air will always be unstable at any altitude wherever the ELR temperature is lower than the MAPR (for example, when the ELR and DALR are equal). The faster the temperature decreases with height, the “steeper” the ELR lapse rate and the more “unstable” the atmosphere can become.

When the ELR-determined temperature falls below the dew point, condensation can occur, provided the correct surface energy and quality of heterogeneous nuclei are available for condensation. Updrafts can move a parcel of air into saturated conditions. Once condensation has begun, it is more appropriate to compare the ELR with the MAPR (or SLR), as the conditions of a rising air parcel will include the presence of water droplets, i.e., saturated conditions prevail [52]. The atmosphere is conditionally stable when the air can rise far enough to become saturated and traverse through conditions where the water vapor could begin to condense. This height is called the condensation level. Unstable air enhances rising motions caused by warming of the Earth’s surface interactions or by traveling cold fronts that displace hot air. Any water condensation or further transformation to ice releases latent heat that may need to be transported down a temperature gradient into the adjoining air, mainly if this happens at temperatures below the equilibrium transformation conditions (aided by recalescence [53,54,55]). Such phase transformations, particularly post-recalescence, may enhance an air parcel’s buoyancy, promoting a faster updraft rate.

### 1.6. PBL

A sublayer of the troposphere, known as the planetary boundary layer (PBL), is a region of the atmosphere where the surface texture influences the temperature distribution, moisture, and wind velocity through turbulent mass transfer. Entropy is generated in the PBL with wind and from friction with the Earth’s texture. This layer also impacts climate, weather, and air quality. The PBL height is generally defined as the altitude of a transition layer where the *air temperature or humidity gradient* is significant within the lowest few kilometers above the surface. The thickness of the PBL depends on the intensity of the surface heating and the amount of water evaporated into the air. The PBL is the major supplier of heat and moisture to thunderstorms. The most dramatic temperature changes occur within the PBL, while the rest of the atmosphere stays at a more predictably uniform temperature lapse rate. At night, with clear skies, the surface cools by radiation, creating a large temperature inversion throughout the PBL. In some cases, the transition between the PBL and the free atmosphere is not well defined; however, the PBL is usually within 100–3000 m of the Earth’s surface. The drier the surface, the higher the PBL. Above the PBL, the horizontal wind speed is more uniform and stronger, as no terrain hinders the winds. *The PBL thus enables vertical flows that can penetrate clouds.* Terrestrial friction causes rotational flow and is the cause of low-pressure development, which can further enable vertical flows. The reported average temperature gradient across the PBL is very similar (but subject to quick changes) to that across the entire troposphere, except for the possibility of inversions. This article discusses additional features of the PBL related to entropy generation. On average, for every 1 °C (1.8 °F) increase in daily maximum surface temperature for a well-mixed PBL, the top of the PBL is elevated ~100 m [7]. The PBL rise is an order of magnitude slower than the tropospheric expansion rate [4]. Cloud bottoms are roughly indicative of the PBL height and are thus considered to remain at a fixed location for the rest of this article.

The rest of this article is organized in a manner first to assess the tropospheric entropy generation rate requirements and the influence of Earth’s surface warming on this requirement. This is followed by a discussion of the type of clouds and other weather phenomena that can meet the high entropy generation rates. Finally, discussions on morphological analogs of cloud patterns are presented to provide possible guidance for the choices a complex system can make for establishing the dominant behavior patterns of its constituents.

## 2. Tropospheric Entropy Generation Rate

The thermal balance between absorbed solar radiation and emitted heat radiation (including absorption and reflection by clouds) mainly determines the temperature of the Earth’s surface. A tropospheric control volume is typically employed for the energy balance [7,8,9,33,56,57,58,59,60,61,62,63,64,65]. A similar tropospheric entropy balance is made in this article, which can additionally model cloud behavior. A control volume approach is considered for the entropy balance calculations, which has provided results consistent with experimental measurements for similar problems in solidification and avian flight organization [17,22,25].

As the radius of the Earth is much greater than the tropospheric height, we can assume that the volume of the troposphere is ~A_Earth_.h. For the model, the troposphere base and top are considered equal making the control volume base area A_Earth_ equal to the Earth’s actual surface area and height, h, equal to the average height of the tropopause. For a control volume that begins at the tropopause and extends to the Earth’s surface, it is possible to write an entropy (S, J/Kg.K) balance as:

Δ**S**_cv_ = (S_in_ − S_out_) + **S**_gen_ (1)

As is known from the myriad of Earth warming data, the overall temperature change in the control volumes is slow compared to the speed of thermal transport and other molecular-scale kinetic transport processes. This allows the model below to approximate that the troposphere is a slow or near-steady-state entropy-generating system. The symbols ρ (kg/m^3^), C_p_ (J/kg.K), are the troposphere’s average density and specific heat (at constant pressure), respectively, t is time, and CV is the control volume. T is the temperature variable, T_av_ is the average temperature of the control volume, and T_E_ is the average temperature of the Earth’s surface. The **s**_gen_ is the entropy generated per unit volume. d**s**_gen_/dt is the entropy generation rate per unit volume that captures all the entropy-generating features from the mass and temperature gradients and phase changes in the control volume [13,17]. The incoming entropy is dS_in_/dt.Δt + entropy transfer from the Earth’s interior via the temperature gradient plus the entropy added by the additional water vapor minus entropy lost from the troposphere by condensing water or ice that falls back to Earth. This is balanced by the entropy accumulation and the entropy out plus the entropy generated. The entropy accumulated and generated in the control volume (CV) is given by:

Δ**S**_cv_ = Ah*ρ*C_p_d(ln(T_av_)) (2)

**S**_gen_ = Ah(d**s**_gen_/dt)Δt (3)

In the equations to follow, Δδ = the change in entropy from water exchange per unit area, i.e., the difference between entropy exchange between the water vapor from evaporation minus the entropy loss from precipitation. Here Λ is the entropy loss by the electrical jets per unit area to the stratosphere plus lightning to the Earth (again per unit area), a catch-all term for entropy loss by thunder and lightning, including that which is lost to the stratosphere or across the CV boundary by electron jets [66]. Here ε_α_ is the average emissivity of the Earth.

In the model below, we have chosen not to parameterize the incoming radiation and instead use the Petela [67] expression for radiation entropy because of the long-wavelength nature of the outgoing radiation (which is typical mainly of only heat transport). A more refined expression is available in reference [33]. The incoming and outgoing entropy into the troposphere by radiation made using the Petela approximation [67] is given by Equations (4) and (5a) (without clouds) and (5b) (with clouds). Appendix A is a scheme to explain groupings of equations to follow.

S_in_ = A_Earth_ψ(ds_in_/dt)Δt (4)

where ψ is the ratio of the square of the distance from the sun to the Earth/square of the radius of the sun ~1.82 × 10^−5^. Without clouds, the entropy leaving the troposphere is

S_out_= A_Earth_(ds_out_/dt)Δt = (4ε_α_ σ A_Earth_T^3^/3)Δt + (4σT_sun_^3^/3)κψΔt + dS_Earth-core_/dt (5a)

Here κ is the percentage of the Sun’s radiation reflected by the Earth’s surface. σ is the Stefan–Boltzmann constant = 5.67 × 10^−8^ J/m^2^K^4^s). An assumption made is that the entropy transfer with solid Earth is small, for electrical jets (lightning). In the presence of clouds, the outgoing entropy by radiation is:

S_out_ = (4A_Earth_Φε_χ_σT_c_^3^/3)Δt + (4σA_Earth_(1 − Φ)ε_α_ T_E_^3^/3)Δt + (4σT_sun_^3^/3)κ_1_ψΔt + dS_Earth-core_/dt (5b)

where κ_1_ is the catch-all average percentage reflected by the Earth and clouds not otherwise captured, e.g., the radiation from Earth and from between clouds that are not mitigated by clouds.

The symbols Φ, ε_ϕ_, and Tc represent the effective cloud coverage, the average cloud emissivity, and the average cloud temperature radiating out of the troposphere, respectively, which establishes the entropy transport out of the tropospheric control volume from clouds. The term ε_ϕ_ also encompasses the influence of the GHG molecules on emissivity.

dS_Earth-core_/dt = [KA_Earth_ (dT_E-core_/dx)^2^/T^2^] is the incoming entropy inside the Earth to the tropospheric control volume. Taking the average thermal conductivity of the soil, even with a high gradient of 30 K/km, gives dS_Earth-core_/dt as a small number compared to the radiative terms, so it will be ignored.

When no clouds are present, the entropy balance per unit area can be written as:

hρC_p_(ln(T)) = (4σψT_sun_^3^/3)Δt − (4ε_α_σT_E_^3^/3)Δt − (4σT_sun_^3^/3)κψΔt + hd**s**_gen_/dtΔt + dΔδ/dtΔt − ΛΔt (6)

Assume dΔδ/dt is the entropy exchange from evaporation and condensation that crosses the control volume boundary located at the Earth’s surface. Suppose the amount evaporated and condensed is the same (not strictly accurate because there is both accumulation in the atmosphere and a difference in the entropy of evaporation and condensation per mass unit of water); this term can be zero or negligible. T_av_ is the average temperature of the troposphere. Λ is the entropy-loss term associated with the catch-all electron jet (like lightning) that crosses the boundaries of the tropospheric control volume (this term will be considered to be small).

Assume dT_av_ /dt = ~dT_cv_/dt = ~dT_E_/dt for the global scale. Noting that ds_in_/dt is a constant (not dependent on the Earth or cloud temperature), the time derivative of Equation (6) yields

ρC_p_ (ln(T_av_))V + hρC_p_(d(ln(T_av_))/dt) = hd**s**_gen_/dt − (4σε_α_T_E_^3^)/3 + dΔδ/dt − Λ (7)

when there are no clouds, and with clouds,

ρC_p_ (ln(T_av_))V + hρC_p_(d(ln(T_av_))/dt) = hd**s**_gen_/dt − (4Φε_χ_ σT_c_^3^)/3 − (4σ(1 − Φ)ε_α_ T_E_^3^)/3 + dΔδ/dt − Λ (8)

where V = dh/dt (the tropospheric expansion rate). Φ, and ε_χ_ are the fraction cloud coverage and emissivity of the clouds, respectively.

Differentiation of Equation (8) and assuming dV/dt = 0 gives Equation (9a). As discussed above, the rate of change in the net water balance is very slow (dΔδ/dt~0) unless the entropy of the water that returns has a very different entropy content than the water that evaporates from the Earth. This can only happen if the water returns as ice or forms at a significantly undercooled non-equilibrium temperature. The corresponding recalescence can have considerably more entropy than from an equilibrium transformation, as is known in other transformations [53,54,55,68]. Regardless the water terms are not considered further.

Assume the second differential for a slowly changing system, e.g., d^2^T/dt^2^ = 0. Note that ρC_p_(d(T_av_)/dt)V/T is small compared to the radiation entropy terms. Further assuming (ds_gen_/dt) is at an extremum implies that (d^2^s_gen_/dt^2^) is zero (or the entire troposphere is at a quasi-steady state at a maximum entropy production rate), so the above equations may be simplified to:

ds_gen_/dt = (ρC_p_/T_av_)d(Tav)/dt + (4σε_α_T_E_^2^/V)dT_E_/dt + C (9a)

Here, C, a constant, is small because the dV/dt, although unknown, is small. Regardless of the dln(T_av_)/dt being small in the simulation without clouds, the term ρC_p_/T_av_ is much smaller than 4σε_α_T^2^/V and Equation (9a) can be approximated to the following expression.

ds_gen_/dt~(4σε_α_T_E_^2^/V)(dT_E_/dt) (9b)

which gives ds_gen_/dt = 5.45 × 10^−5^ W/m^3^K for the current Earth conditions (T_E_ = 287.5 and dT_E_/dt = 5.7 × 10^−10^ K/s).

Equation (9b) could be seen to imply no new entropy generation demands in the troposphere for conditions of zero warming rate; however, warming has a diurnal and seasonal rate component in addition to the global trend. Equation (9b) is, therefore, more appropriately recast for the additional s_gen_ produced per degree of warming as:

((ds_gen_)/dt)/(d(T_E_)/dt) = 4σε_α_T_E_^2^/V (9c)

**Figure 2:**
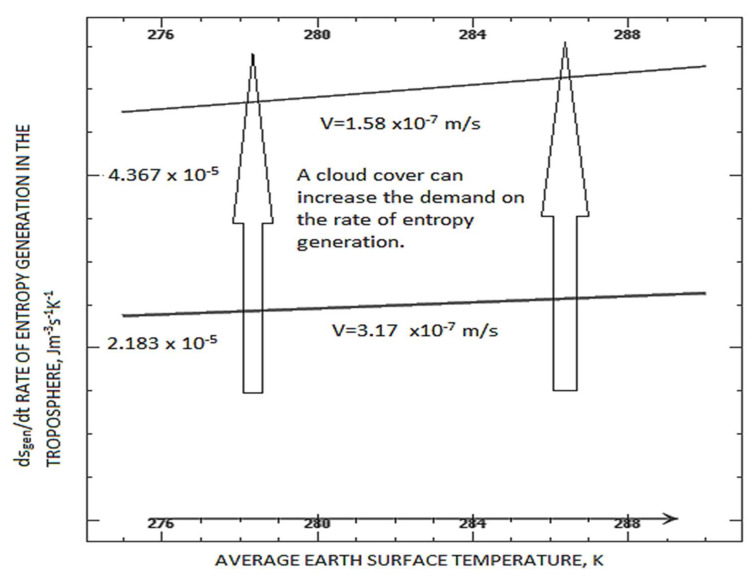
Entropy rate generation per unit volume vs. Earth’s temperature (K) at the present rate of surface warming and assuming no clouds and dT_E_/dt = 0.018 K/year. The Stefan–Boltzmann constant σ = 5.57 × 10^−8^ W/m^2^K^4^, emissivity ε_α =_ 0.85, tropospheric expansion velocity V = (1.58 and 3.17) × 10^−7^ m/s. Cloud formation can increase or mitigate the demand on the entropy generation rate (vertical arrows indicate this).

The entropy generation rate as a function of the surface temperature at the current rate of warming for the tropospheric expansion rate of 5 m/year is shown in Figure 2. Assuming h = 20 × 10^3^ m and the surface area of the Earth is 5.1 × 10^14^ m^2^ yields the total entropy generation rate for the troposphere equal to dSgen/dt equal to 5.559 × 10^−14^ J/(K.s) for the current temperature of 287.5 K (i.e., ds_gen_/dt = 5.45 × 10^−5^ W/m^3^K). This entropy generation dSgen/dt calculated with the low complexity model is close to the value of (6.4–6.5) × 10^−14^ J/(K.s) reported by Wu and Liu [33].

Similarly, with clouds and precipitation, the entropy generation rate can be inferred from Equation (8) to be:

ds_gen_/dt~[4Φσε_χ_Tc^2^dT_E_/dt + 4σε_α_(1 − Φ)T^2^dT_E_/dt]/V − (−dΔδ/dt) + dΛ/dt (10)

The average Earth’s temperature in 1900 was ~13.6 °C with 300 ppm atmospheric CO_2_ concentration, and in 2020, it was 14.6 °C with 370 ppm, which gives the entropy generation rate density per ppm CO_2_ as approximately equal to 6 × 10^−9^ J/(m^3^.K.s.ppm(CO_2_)).

## 3. Discussions

Equations (9a–c) and (10) indicate that the demand for a higher entropy generation rate directly results from a higher Earth surface temperature. This is plotted in Figure 2 (a plot of Equation (9) for an Earth emissivity of 0.85). It will be noted below that the model seems applicable to diurnal changes, seasonal changes as well and slower global warming effects. A quasi-steady-state approximation is valid for both time scales. In complex systems, there is little guidance on whether the dominant phenomena are at a small scale or a larger scale. In reference [22], it was shown that the scale of dominant phenomena could increase or decrease with an increasing rate of entropy generation per unit volume. Although it is not possible to isolate every possible entropy generation phenomenon in the troposphere, the fact that the rate of atomic vibrations or two-phase transfer of atoms across an interface is substantially faster than most climate phenomena of wind rate or rain rate (i.e., except perhaps for lightning bolts) is an indication that the use of the quasi-steady-state approximation for Equation (9) is reasonable.

Cloud formation alters the demand for the entropy generation rate. This is inferred by comparing Equations (9) and (10). However, the influence of clouds on the demands for entropy generation rate is related to the relative emissivity in Equations (9) and (10). The emissivity of Earth’s surfaces ranges between 0.6 and 1 [58]. Surfaces with an emissivity of less than 0.85 are typically found in dry areas closer to the equator. The emissivity of clouds increases with increasing cloud thickness [58]. Low-level clouds have an emissivity close to 1. The ε_a_ increases in the polar regions. Altocumulus clouds have a mean emissivity of about 0.8. The emissivity of high-altitude cirrus clouds ranges from 0 to 1, with a mean of 0.35 [8]. The emissivity of water (oceans) is ~0.95. Thus, new cloud formation over the oceans may not significantly alter or even reduce the entropy production rate demand when compared to a situation without clouds because the emissivity of the underlying water is like that of clouds—unless there is a net loss of entropy from water exchange in the troposphere in the presence of clouds. On the other hand, the emissivity of clouds appears to be larger over land, which, based on Equation (10), would make the presence of clouds increase the demand on the rate of entropy generation. A rigorous sensitivity analysis for the uncertainty in the output of the mathematical model where it can be divided and allocated to different sources of uncertainty in its inputs was not performed, because several terms are dominated by a single physical phenomenon for the complex tropospheric system. Regardless, the sensitivity of Equation (10) to cloud coverage and emissivity change is inferred to be high—this is why the conclusions offered in the article can track both diurnal and long-term changes.

If the troposphere attempts to maximize the entropy generation rate, a comparison of Equations (9) and (10) indicates that cloud appearance could be favored, provided adequate atmospheric moisture is available. This is particularly true over land masses where the emissivity of the surface is low compared to high-emissivity clouds. However, it is not the same over large water masses, as the difference in the emissivity of water and the low-lying thick clouds is not significant. The radiative impact of low-level clouds is controlled by their liquid water content (LWC). Although not rigorously proven with the arguments made thus far, this could indicate that large-droplet water-containing clouds could have a higher emissivity than smaller-sized ones. Therefore, over water masses, there is less propensity to form additional clouds with additional warming, unlike the situation over land masses. Still, there is more propensity for the moisture-laden atmosphere to form clouds over land. In an experiment performed on the seas, fewer low-lying wet clouds were observed when sulfate particles (nuclei) in the atmosphere above the water were reduced [59]. This observation is in line with the implication that the emissivity of cloud formation on the seas is not particularly important to the tropospheric entropy generation rate, as discussed above.

The principle of MEPR [10,12,13,14,15,16,17,18,19,20,21,22,23,32] suggests that the tropospheric system should evolve towards the highest entropy generation rate condition while searching for a steady state, or it could spatially and temporally oscillate in the type of patterns like a BZ reaction while attempting to establish a state that maximizes the rate of entropy production [69,70,71]. In the following discussions, it will be shown that the conditions for increased-intensity rainfall and other such events could be related to both an increasing driving force and increased high-intensity entropy generation rates in specific cloud patterns. The increased driving force is directly related to the amount of water vapor, undercooling, and updraft velocity, all promoted by warming. These conditions increase the tropospheric demand for a high entropy generation rate.

There is a growing recognition that the patterns of clouds and condensation patterns today are entirely different in scale and intensity than previously encountered [72]. Measurements suggest that an increase in the number of cumulus clouds and thunderstorms is noted with an increase in Earth’s surface temperature [56,72]. An increase in the surface temperature increases the number of lightning strikes [51,57]. In the following discussion, we first examine the effect of clouds on the entropy generation rate with a similar entropy balance model presented in the previous section. Then we examine the role of clouds to assess the direction of typical weather changes that are anticipated with Earth’s surface and tropospheric warming. Specific cloud types are examined to assess the entropy generation in each type. Wherever possible, we draw verification for the results from published observations from specific cloud identification.

### 3.1. Entropy Generation in a Cloud without Significant Vertical Development

For clouds, an entropy balance in a control volume, defined by the top and bottom boundaries of a cloud, can be written as

ξρC_p_(ln(T_c(av)_)) = (4σψT_sun_^3^/3)Δt + (4λε_α_ σT_E_^3^/3)Δt − (4ε_χ_.σT_cb_^3^/3)Δt − (4ε_χ_κ σT_c_^3^/3)Δt + ξ.d**s**_gen_/dt. Δt − dΔδ/dt (11)

T_c(av)_ and Tc are the average and cloud-top cloud temperatures, respectively. The terms ξ, and T_cb_ are the cloud thickness and the temperature of the cloud base, which for low-lying clouds could be approximated to be the temperature at the PBL height, a constant from known observations. On the RHS, the first term is incoming entropy, the second is outgoing entropy generated by the radiation from the Earth and mitigated by clouds and the tropospheric atmosphere, and the following two terms are the entropy amounts leaving the cloud towards Earth and the stratosphere, respectively. These include the term κ, which considers the reflectivity of radiation between the Earth and cloud bottoms. Here, λ is the correction factor for the pass-through radiation in thin transparent clouds.

Differentiation of Equation (11) with respect to time and if the reflectivity terms cancel or are negligible gives:

ρC_p_ln(T_c(av)_)V_c_ + hρC_p_(d(ln(T_avc_))/dt) = ξd**s**_gen_/dt + 4λε_α_ σT_E_^3^/3 − (4ε_χ_σT_cb_^3^)/3 − (4ε_χ_σT_c_^3^)/3 − ξΔ*f_v_*ds_water_/dt (12)

Here Δ*f_v_* ds_water_/dt is the rate of entropy loss from the cloud because of rainfall. T_avc_ is the average cloud temperature. A_c_ is the cloud area projected normal to the Earth’s surface, *r_v_* is the mixing ratio of water vapor, and Δs_water_ is the entropy difference from vapor to liquid at the equilibrium transformation temperature at the altitude pressure.

If V_c_ = d(ξ)/dt = 0, i.e., there is no cloud thickening, and if the second term on the LHS of Equation (12) is small, the entropy generation rate per unit volume is given by

ds_gen_/dt = [(4ε_χ_σT_cb_^3^) − (4σψ T_sun_^3^) + (4ε_χ_κ_1_σT_c_^3^)] – [(4λε_α_σT_E_^3^)]/3ξ (13a)

ds_gen_/dt~[(8ε_χ_σT_c_^3^ − 4ε_α_σT_E_^3^)]/3ξ ~ [(8ε_χ_σT_c(av)_^3^ − 4ε_α_σT_E_^3^)]/3ξ (13b)

when there is no rainfall, and

ds_gen_/dt~[(8ε_χ_κ_1_σT_c(av)_^3^ − 4λε_α_σT_E_^3^)]/3ξ + [ξΔ*f_v_* ds_water_/dt]/ξ (14)

when the rate of rainfall is non-zero. Here T_c(av)_ is approximately ½(T_c_ + T_cb_).

ds_gen_/dt~[[(8ε_χ_κ_1_σT_c_^2^ − 4λε_α_σT_E_^2^)]/3] + [ξΔ*f_v_* ds_water_/dt] + [ρC_p_(ln(T_avc_))V_c_]SLR/ΔT (15a)

Here κ_1_ is the average catch-all percentage of radiation the Earth and clouds reflect, λ is the correction factor for the pass-through radiation in thin transparent clouds, and ΔT is the temperature difference between the cloud’s bottom and top and

ξ = ΔT/SLR(15b)

**Figure 3:**
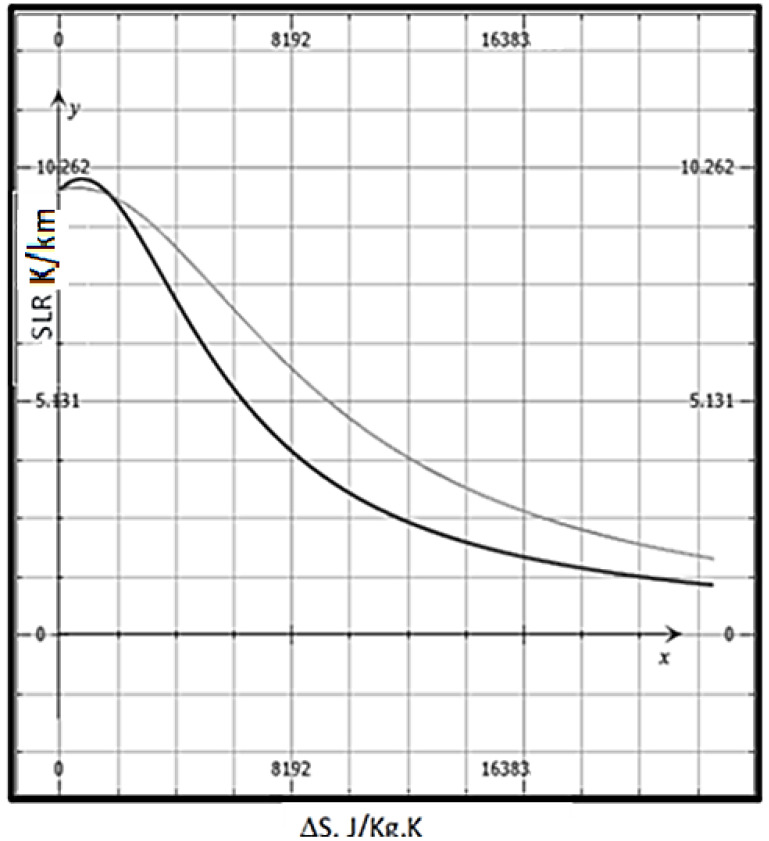
A plot of SLR (K/km) as a function of Δ*S* (J/Kg. K). The heat of vaporization Δ*H* = 2.501 × 10^6^ Jkg^−1^ is assumed constant, C_p_ = 1005 JK^−1^kg^−1^, C_pv_ = 1850 JK^−1^kg^−1^, the gas constant for water vapor R_v_ = 461 JK^−1^kg^−1^. The r_v_, is the mixing ratio of water vapor (mass ratio). The thick line corresponds to high water content, r_v_ = 0.038 (3.8%). The thin line is for low water content, r_v_ = 0.01 (1%).

The saturation air lapse rate (SLR) is given namely [61,62]

(15c) $$SLR=-g(\frac{(1+(\Delta {S/R)r_{v}\varepsilon )}^{\;}}{(Cp+({\Delta S}^{2}{/R)r_{v}ɳ)}^{\;}})$$

where Δ*S* is the entropy change on condensation, *r_v_* is the mixing ratio of water vapor, g is the acceleration due to gravity, *C_p_* is the specific heat at constant pressure of dry air, and *r_v_* is the water-vapor mixing ratio. We can approximate ΔS~ΔSv~ΔH/T the entropy of vaporization; *R* is the gas constant for dry air (universal gas constant divided by the molar mass), ɳ is the ratio of the gas constants for dry air and water vapor, ΔH is the heat of vaporization, and *T* is the equilibrium temperature. The subscripts d, v, and l indicate dry air, water vapor, and liquid water, respectively. Equation (15c) reduces to the DALR for dry air, i.e., when *r_v_* = 0, and approximates the MALR at saturation. As the temperatures decrease with altitude, Δ*S* will correspondingly increase with a drop in temperature, i.e., with altitude, assuming that the heat of vaporization is not a function of temperature. For *r_v_* less than 0.03, this Equation, although nonlinear with Δ*S*, can make the theoretical SLR less steep and thus increase the thermodynamic driving force for precipitation. The water vapor mixing ratio is in g kg^−1^. The water vapor mixing ratio, *r_v_*, is typically at most about 38 g kg^−1^ or 0.038 kg.kg^−1^. A simplified SLR for low saturation that is sometimes employed is −g/C_p_(1 − 0.85r_v_); however, this simplification does not capture the high undercooling (higher ΔS) that can yield the correct SLR, as shown in Figure 3. Note that this ratio is well above the number two, which is an indicator of a smoothly bounded diffuse interface [17].

Figure 3 shows that a lower SLR is enabled by a higher *r_v_*, i.e., the water content. Thus, enhanced evaporation and higher tropospheric water content (from the increasing temperature enabled by climate change) can always lead to more condensation. Measurements have shown, however, that the undercooling does not necessarily keep increasing with height above the cloud base but tends to diminish as the T_c_ level rises toward the tropopause [2]. The difference between SLR at saturation conditions (an approximation for the MAPR) and the dew-point temperature at any altitude is the undercooling (driving force). The higher water content in the troposphere (or sometimes called hydrosphere) increases the precipitation, leading to more water per unit volume that can coalesce and drop to the Earth’s surface.

The effective cloud droplet radius is larger over oceans than over land by about 15% to 20% [9]. Consequently, maritime (sea) clouds tend to have fewer water droplets than continental (land) clouds. Most maritime clouds have droplet concentrations between 100 drops/cm^3^ and 200 drops/cm^3^. Land clouds have much higher droplet concentrations, up to around 900 drops/cm^3^. The effective particle radius ratio of condensed water droplets and ice particles in clouds over oceans differs significantly from that of land. For oceans, it is around 14 μm and 25 μm, respectively. In contrast, the two sizes are the same over land, suggesting that the land clouds involve vapor-to-condensed phase transformation occurring at much higher altitudes, thus also suggesting that the entropy generation rate could be much higher with land clouds because of higher undercooling from stronger updrafts, i.e., more entropy generation is possible per unit volume for land clouds.

Assuming that the MAPR is approximated by the SLR, Equation (15a) can be written as;

ds_gen_/dt~[[(8ε_χ_κσT_c_^2^ − 4ε_α_λσT_E_^2^)]/3] + [ξΔ*f_v_* ds_water_/dt] + [ρC_p_ln(T_c(av)_)V_c_]SLR/ΔT (15d)

Or, when the first and third terms of Equation (15d) are zero, the Equation reduces to

ds_gen_/dt~[(ξΔ*f_v_* ds_water_/dt)SLR/ΔT] (15e)

which is also zero when there is no rainfall.

S_water_~S_vpi_ is the entropy change for transforming vapor into water or ice. Note that the moist lapse rate of near-saturation air is a function of the entropy of condensation, which, if it happens at a lower temperature than the equilibrium at any altitude, will lead to a higher entropy of transformation, provided the enthalpy of transformation is unaffected by the undercooling.

It can be noted from Figure 3 that the SLR increases with a lower Δ*S*, which, e.g., for vapor-to-liquid transformation compared to vapor-to-ice transformation, could lower T_E,_ and produce rain compared to high stratus with small vertical development or cirrus clouds (ice-containing). Stratus and cirrus clouds are sometimes spread out in patches, with ample sky breaks between them. Stratus clouds are thick, gray clouds that look like fog with a base above the ground. Stratus clouds often produce light, drizzly rain or snow, especially from a nimbostratus cloud. These typically are low-altitude clouds. Clouds may be able to remain stratus-like once they are formed and grow or join other horizontal clouds forming at similar altitudes.

Note that d_sgen_/dt cannot be less than zero. Although the second and third terms in Equation (15d) can influence the entropy generation, the grouping of the first term on the RHS of Equation (15d) must be positive. Cloud types such as status and cirrus with no vertical development are thus limited in their ability to generate entropy. Although horizontal stratus clouds often produce light, drizzly rain or snow, especially when nimbostratus, the thin cloud formations do not easily cool the Earth’s surface, and consequently T_c_ < T_E_.

We can thus think of the stratus and cirrus horizontal clouds as representing equilibrium clouds—because the entropy-generating term is minimal especially when V_c_~0 i.e., when there is no rain (the role of V_c_ is made more apparent in the next section). Therefore, for clouds with no vertical development and no change in the rainfall rate, the entropy generation per unit volume rate can only be enhanced in the troposphere by progressively thinning clouds (low ξ). Should they form, cirrus and stratus, i.e., horizontal clouds, must thin (become wispy and disperse) when the demand on the rate of entropy increases because of the warming that these clouds enable. For small entropy generation in horizontal clouds with limited vertical development, the following bifurcation condition at least must be met when there is no rain and if LSR~0:

2ε_χ_κ_1_T_c(av)_^3^/ε_α_λT_E_^3^ > 1 (16)

Equation (16) is thus the approximate criterion for atmospheric breakdown for inclement weather because as we will see in the next subsection (Section 3.2), entropy-generating clouds will quickly develop vertical velocities (updrafts) to enhance the entropy generation rate whenever required provided there is moisture available.

### 3.2. Entropy Generation in a Cloud with Vertical Development

In contrast to equilibrium horizontal clouds, a significant amount of entropy generation is possible by clouds that show vertical development, as shown below. Cloud evolution processes are dynamic phase-change processes where significant entropy changes can manifest across transformation zones (diffuse interfaces). Thick cumulus clouds are bright white and look like big puffs of cotton. These clouds, sometimes called thunderheads, form into the shape of an anvil, which is a sure sign of a storm! Heavy thunderstorms and even tornadoes are associated with this type of cloud (a tornado is a rotating column of air connected to a cumulonimbus cloud). These clouds can be so huge that their bases start only 300 m above the ground with a top of 12 km. Updrafts build clouds to heights of up to ~12 km or more, i.e., to the tropopause level at mid-latitudes.

Vertical movement of air near the Earth’s surface occurs on account of the buoyancy of air [21,46,63]. The vertical structure of liquid water content in shallow clouds is obtained accurately from dual-wavelength radar observations [45] and is almost linear from the bottom to the top. This vertical movement of air leads to the upward transport of significant amounts of energy. The lapse rate in the PBL can also be affected by upward air movement. The moist adiabatic lapse rate is ~(−4–7) K/km. The rising moist air thus cools only about two-thirds as fast as dry air (DALR), whereas the overall ELR can be steeper [7,43,49,50,72,73]. This difference and any increase in the moisture content impacts the vertical velocity and phase transformation rate of a moist air parcel and, thus, the cloud formation rate and amount. As the warm, moist air cools while it rises, the moisture condenses into water droplets, releasing latent heat (particularly as undercooled droplets recalescence while still in an updraft), thus warming the rising air. When this happens, it is buoyant and consequently rises faster, i.e., with the moist air cooling more slowly compared to dryer air. This allows moist, warm air to rise much longer and reach significant heights. A more significant temperature difference with the surrounding air is also maintained when the rise is rapid (i.e., before droplets coalesce and start falling towards the Earth as rain, hail, or snow—a feature that can lead to increased condensation during the buildup of a thundercloud). Additionally, positively charged particles are moved to the top of the cloud, and negatively charged particles are moved to the bottom of the cloud a process that produces entropy.

Vertically developing clouds produce new entropy from transforming vapor to condensed matter at non-equilibrium temperatures. This is like the diffuse interface mechanism of undercooling for entropy production [17,45]. Cumulus clouds with vertical development increase with warming [51,56,57,72]. Significant vertical velocities can lead to the formation of cumulonimbus clouds over land and oceans. However, maritime clouds may have to be blown over land to promote more (severe) taller clouds with significantly enhanced precipitation. This could explain why the warming of the Earth is leading to more precipitation over land masses than the ocean.

Recalescence heating can also aid entropy generation [13,53,54] and even water phase separation [40]. Ice formation from vapor is a mechanism for high entropy generation rates. Such conditions can increase the albedo further accelerating the increase in the Earth-surface and tropospheric temperatures. Clouds are regions where entropy generation occurs from this phase change from vapor to liquid or solid H_2_O. A mixed layer can form to a height where the static stability of the air forms a barrier to thermally induced upward motion, particularly as the size of the condensed phase increases. This occurs practically daily over the arid areas of the world, where the limit to upward mixing is often the tropopause itself.

When the temperature of moist air falls below the dew point, precipitation becomes possible, subject to any nucleation difficulties (initially, condensed droplets are micron-size and support the upward motion, and when coagulated, they can reach mm size and fall to the ground). The height at which the moisture initially condenses, however, caught in an updraft, can be observed visually as the height of the bases of clouds (most likely, these are contiguous with the PBL height). This structure usually marks the location of the thunderstorm updraft and portends the development of tornadoes. The total water mixing ratio in a rising moist parcel can decrease when the condensed phase (rain and ice) grows large, thus reducing the driving force for further condensation or updrafts. However, as the rain falls through unsaturated air, evaporation could occur again, increasing the total water mixing ratio and the entropy of the air. This mixture of water droplets in condensed saturated, condensed, or partially saturated air is like a diffuse interface, i.e., a region of two or three phases that are mixed intimately.

The cumulonimbus cloud formation patterns associated with large amounts of atmospheric water and updraft are promoted by a higher Earth surface temperature and a corresponding increase in the demand and production of the entropy generation rate. Undercooled droplets that nucleate and recalesce rapidly can cause an inversion in the lapse rate, which, in the presence of a strong updraft, could make the LSLR and MALR generate the conditions for stronger precipitation. Severe thunderstorms form as warm, moist air ahead of a cold front is forced to rise into unstable air or along squall lines with microbursts, with strong downward winds that can exceed 100 mph. Strong vertical winds may cause the storm to rotate—a possible precursor to tornado formation capable of producing large hail, strong winds, flash floods, and tornadoes.

The formation of thick, vertically developing clouds is a feature of severe weather. When there is vertical development, Equation (12) can be examined again with the velocity term. If V_c_ = d(ξ)/dt is non-zero, and if dT_cb_/dt = 0, i.e., the cloud base temperatures are constant,

ds_gen_/dt~[ξ dΔ(*f_v_*(s_vpi_)/dt)] + [ρC_p_ln(**T_c(av)_**)V_c_]SLR**/**ΔT (17)

where the entropy generation rate increases with vertical velocity and increases with *f_v_*, i.e., with altitude.

Entropy is generated in a density gradient inside a diffuse interface. Sekhar [13] described diffuse interfaces or mixed-phase regions as regions of mixed-mode transformations in a control volume where considerable new entropy is generated at various rates. Following the techniques proposed by Bensah et al. [17,45] for estimating the entropy generation rate in diffuse interfaces for liquid–solid transformations, it is possible to model clouds as diffuse interfaces with a gradient of the fraction of water from the top of the cloud to the bottom of the cloud. The diffuse-interface methods discussed in reference [45] are used to calculate the entropy generation rate for a cumulus or vertically developing cloud. A linear density gradient is assumed based on the linear fraction of water content. The measurements inside clouds for the density gradient show an average linear gradient [2]. The water fraction is assumed to decrease linearly from the cloud base to the top. Thus, ds_gen_/dt becomes a function of ξ, V_c_, and SLR. The loss in work potential (free-energy dissipation) in a vertically unstable cloud is the entropy generation rate multiplied by the average temperature. The cloud thickness ξ is the thickness of the diffuse interface. T_av_. dsgen/dt represents the rate of work potential loss for an average value of *f_v_* in the diffuse interface. Following the derivations shown in references [17,45,55,74], the rate can be written as

ds_gen_/dt = ∆*ρ*_*k*_*f_v_* V_c_^3^/2ξ ^2^SLR (18)

where ∆*ρ*_*k*_ is the density difference between the condensed phase and the vapor phase. Alternately, again from references [17,45], it is possible to write the ds_gen_/dt (J/m^3^.K.s) in the following form:

ds_gen_/dt = V_c_*f_v_*Δh_vl_SLR/T_cb_T_cT_ (19)

The vertical velocity, volume fraction of water, *f_v_* in clouds, SLR, enthalpy of precipitation, and cloud envelope temperatures determine the entropy generation rate per unit volume in clouds.

Note that Δh_vl_ is volumetric, e.g., in the units of J/m^3^. The typical values of *f_v_* for various cloud types are shown in Table 1.

Equations (9) and (18) gives

A_Earth_h[4Φσε_χ_T_c_^2^dT_E_/dt + 4σε_α_(1 − Φ)T_E_^2^dT_E_/dt]/V = Acξ[∆*ρ*_*k*_*f_v_* V_c_^3^/2ξ ^2^SLR] (20)

which reduces to

A_Earth_h (4σε_α_T_E_^2^/V)(dT_E_/dt) = Acξ[∆*ρ*_*k*_*f_v_*V_c_^3^/2ξ ^2^SLR] (21)

The cloud updraft velocity is given by Equation (22).

V_c_^3^ = (2A_Earth_h SLR(4σε_α_ T_E_^2^/V)(dT_E_/dt))/(Ac∆*ρ*_*k*_*f_v_*) (22)

V is the tropospheric expansion rate, and V_c_ is the cloud updraft rate. A_Earth_ and A_c_ refer to the area of the Earth and cloud, respectively. Plugging in typical values of the surface areas, a cloud Earth/cloud area ratio of 0.66, and the parameters used in Figure 1, with *f_v_* = 3 × 10^−6^, SLR = 4 × 10^−3^ K/m, and a tropospheric height of 20 km, Equation (22) yields

V_c_ (m/s)~(0.05ξ)^0.33^ (23)

where ξ is in meters.

The amazing order-of-magnitude fit with the velocities shown in Table 1 is an indicator that the diffuse-interface model used is predictive.

**Figure 4:**
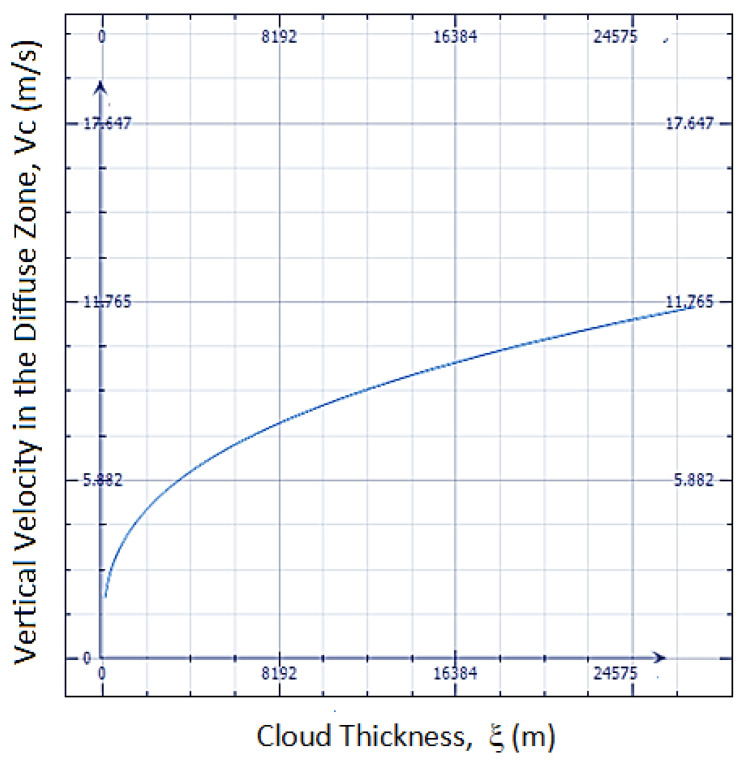
The predicted vertical updraft velocity for a saturated cloud developing vertically with the parameters used in Figure 2, namely, V = 5 m/year and dT_E_/dt = 0.018 K/year. The Stefan–Boltzmann constant σ = 5.57 × 10^−8^ W/m^2^K^4^, emissivity ε_α_ = 0.85. The current rate of Earth warming is dT_E_/dt = 5.7 × 10^−10^ K/s.

Equation (22) indicates that V_c_ will increase with dT_E_/dt (i.e., warming will increase the severity of the weather by enhancing updrafts, which leads to severe weather). Equation (23), plotted in Figure 4, is the calculated updraft velocity from the diffuse-interface formulation. For a near-constant cloud fraction A_Earth_/Ac and identical ∆*ρ*_*k*_ and *r_v_ for the cloud as it evolves*, any increase in SLR and cloud thickness will increase weather severity, i.e., the cloud can evolve, e.g., into a hail-spewing thundercloud. When T_E_ decreases because of low-lying fixed-latitude clouds, shielding the sun’s warming, or if the SLR decreases, V_c_ will decrease. This situation also simulates the final stage of a thunderstorm, where a decrease in the cloud thickness **ξ** will reduce the entropy rate production. An approximation where the vertically developing V_c_ > ~0.1 m/s could be a measure for entropy-producing clouds becoming dominant over other entropy-production phenomena like winds. If the entropy-generating high-water-content cloud moves horizontally, typical movement from maritime regions to regions of high surface temperatures, hurricanes (or tornadoes over land) and lightning can manifest. Finally, in the last stage of a heavy rain-or-hail-fall cumulonimbus cloud, the system attempts to approach a new steady state and leaves behind wispy cirrus clouds. The equations for the entropy generation rate from references [17,45] can also be used to extend Equation (19) in the following manner.

ds_gen_/dt = V_c_*f_v_*Δh_vl_SLR/T_cb_T_cT_ = {[(1/SLR)dΔ(*f_v_*(s_vpi_)/dt)] + ρC_p_ln(T_avc_)V_c_/ΔT}SLR (24)

which can be used for assessing the mature stage of a thunderstorm. Numerically, Equations (18) and (24) predict a correct order of magnitude of the entropy generation rate per unit volume to match the demand amount shown in Figure 2 for typical cloud thicknesses and water content.

V_c_ = [ξ dΔ(*f_v_*(s_vpi_)/dt) + C_p_ln(T_avc_)V_c_/(*f_v_* Δh_vl_)][T_cb_T_cT_/ΔT] (25)

**Figure 5:**
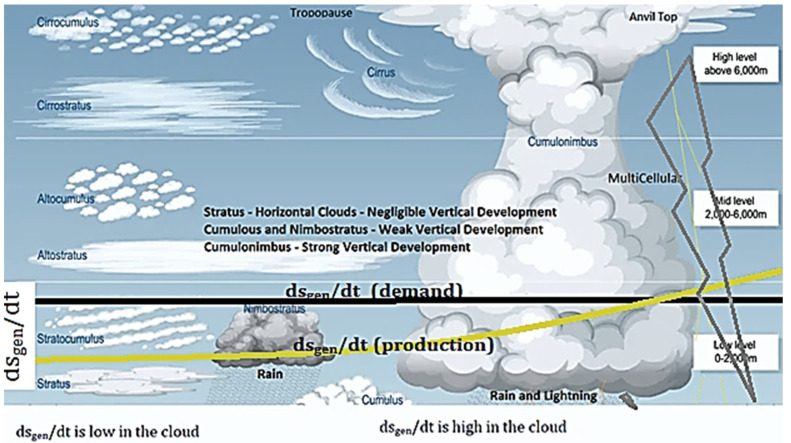
The demand for tropospheric entropy generation and entropy generation rates available from clouds are schematically illustrated. The expected clouds and related weather phenomena are also illustrated. Stratus clouds are closer-to-equilibrium clouds (with zero to low entropy production rates). However, they can aid warming. In contrast, cumulus and nimbus clouds are non-equilibrium clouds with an entropy generation rate in the cloud, which increases with the vertical updraft velocity. Multicell development, lighting, and swirls add to the rate.

**Figure 6:**
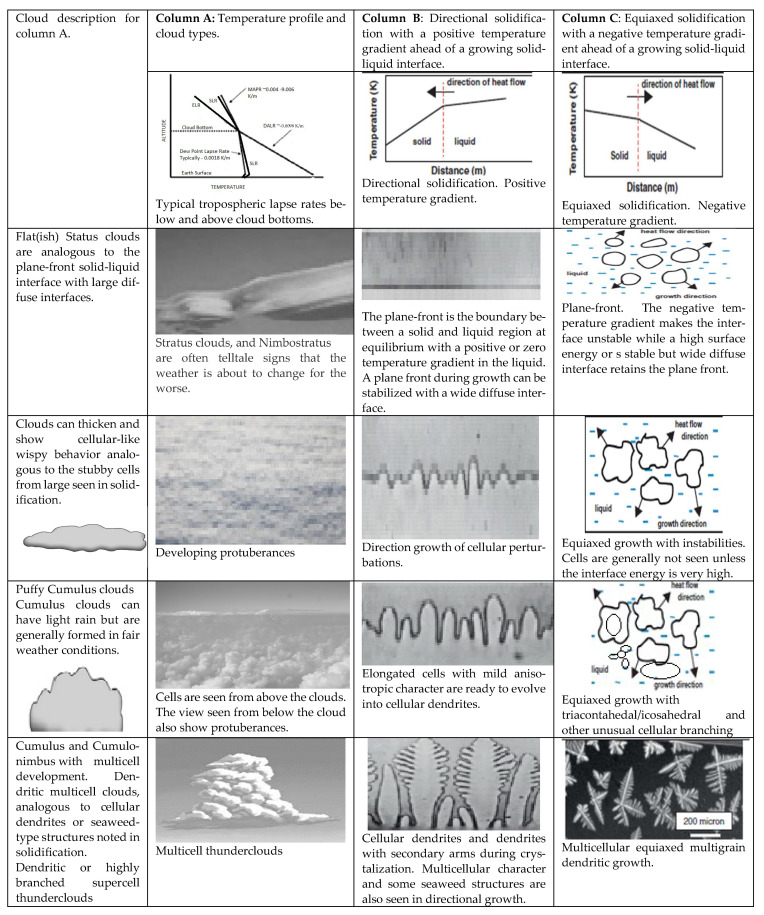
A pictorial analogy of the pattern formations in the diffuse-interface regions of clouds (Column A) and solidification microstructures (with a positive (Column B) and negative (column C) temperature gradient in front of the solidifying interface). Adapted from references [74,75,82,83,84,85].

Finally, we can also estimate when stratus clouds evolve into cumulus-stratus clouds, i.e., the onset of vertical development with curved perturbations (see Figure 5 and Figure 6) with the following equation:

[ξ dΔ(*f_v_*(s_vpi_)/dt)] + [ρC_p_ln(T_avc_)V_c_]SLR/ΔT = ∆*ρ*_*k*_ *f_v_* V_c_^3^/2ξ ^2^SLR (26)

Lightning is yet another mechanism for enhancing the rate. Lightning and thunder are rapid discharges (sparks) occurring in mature thunderstorms within clouds or between clouds and ground, clouds, and stratosphere. This is an additional mechanism to generate and transfer entropy at high rates along with the energy transfer across the tropospheric control volume boundaries. The lightning strike heats the air to high temperatures with explosive expansion, producing a shock wave and thunder. 

Figure 5 illustrates the demand and production features for the entropy generation rate and associated cloud formations. In thunderclouds, a significant downdraft begins when precipitation starts, and electrical phenomena for energy transport can arise. The charge at the bottom of the cloud is large enough to produce potential differences of even 100 million volts between the cloud and the Earth—much bigger than the 0.4 million volts from the “sky” to the ground in a clear atmosphere [75,76]. The top of a thunderstorm has a positive charge, and the bottom mostly has a negative charge. When the difference in potential exceeds the discharge threshold (caused by the separation of charges), powerful lightning occurs, accompanied by thunder. The potential gradient (volts) causes entropy generation when a charge flow or discharge is causing a significant entropy generation spike (note this term was ignored earlier when developing Equations (9) and (10)).

For extreme weather assertion based on an increasing demand for entropy generation rate, a relationship with the parameters that encourage mature thunderstorms to further entropy rate generation is required. These could involve creating multicells in thunderstorms. Figure 5 shows a schematic of such a relationship. Sometimes, the atmospheric conditions encourage vigorous new cell growth in thunderstorms—they form so fast that each new cell develops further upstream, appearing as though the thunderstorm cluster is stationary or moving backward against the upper-level wind. Forming a multicell is likely to accelerate the entropy generation rate per unit volume.

### 3.3. Intense Weather and Entropy Generation in Complex Systems

A complex multi-component system like the troposphere is likely to dissipate more energy at larger scales when the demand on the entropy generation rate increases, resulting in more significant large-scale entropy production mechanisms that are offered up by the rapid wind gusts competing with vertically developing cloud formations for maximizing the rate of entropy generation. A complex system addresses the spatially large and spatially small temperature, pressure, and chemical potential gradients [15,18,21,23,28,29,30,77] by selecting shapes (patterns) or energy dissipation processes that maximize the optimal entropy generation mechanism. The optimization process establishes the system’s self-organized behavior for establishing patterns and shapes. Such optimization also happens in other complex systems [11,12,13,14,15,16,17,18,19,20,21,22,28,29,30,32,33,34,45,69,70,71,77]. A control volume’s entropy production efficiency is most likely not optimized for free-energy dissipation, as it adjusts the scale at which entropy is produced. A change in large-scale entropy production mechanisms can lead to an increase or decrease in smaller-scale entropy production mechanisms.

The vertical velocity is related to the droplet concentration in clouds [78,79,80,81]. The vertical velocity and liquid-water content of a cloud also correlate to the height of a cloud from its base. When vertical wind shear is weak, thunderstorms can quickly disappear after maturity. However, as vertical wind shear increases in the atmosphere, conditions become more favorable for thunderstorms to take on new morphologies and last longer for highly buoyant parcels of rising saturated air with accompanying lightning and thunder. Clouds often become multicellular or even develop into a supercell cloud with a tornado. Multicellular thunderstorms are a “group” or “family” of single cells at various stages of their life cycles. 

**Figure 7:**
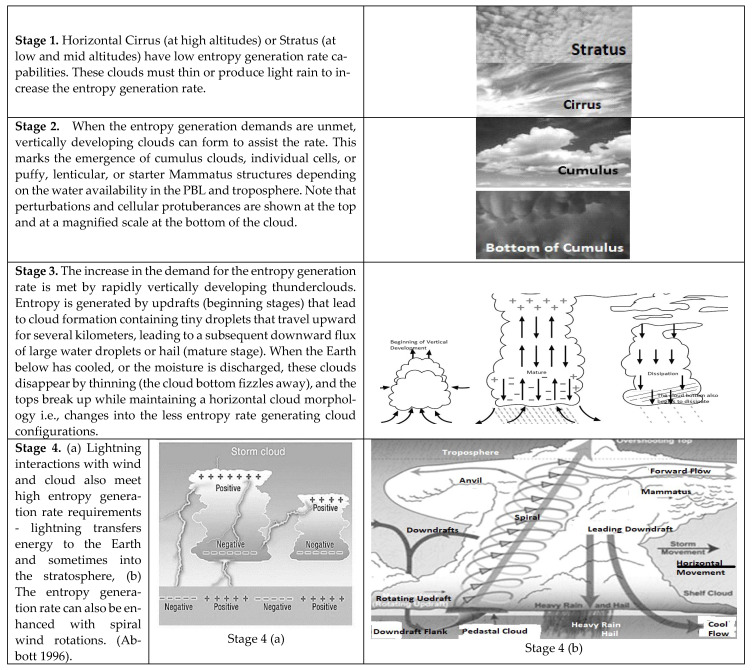
Stages of entropy-generating cloud evolution with increasing surface temperatures that finally lead to severe weather. Adapted from references [46,76,86,87,88].

Multi- and supercell behavior with strong horizontal winds that interact with vertically growing clouds leads to swirling tornados, hurricanes, and lightning—ways to keep the entropy production at a high rate by discharging energy across the control volume boundaries from an entropy-generating control volume. Other ways of forming multicellular clouds are also possible. A supercell cloud can have multiple smaller distinguishable cells. One example where such tropospheric features are noted is when continental cold, dry air penetrates an adjacent relatively warm ocean, creating updrafts. The updraft leads to cloud formation, which looks like a river in the sky, approximately parallel to the direction of the wind. Such rivers develop into three-dimensional opened cells. Because of intense turbulence in the PBL, which creates updrafts, a cumulus shape can form that can further magnify. The dissipating stage of a thunderstorm is noted to begin when the downdrafts in the cloud become strong enough to prevent updrafts. This leads to the last stage of a thunderstorm, where it weakens and dissipates. The storm dies out with light rain as the cloud disappears from bottom to top (see Figure 7), leaving behind horizontal or wispy cirrus morphologies.

A pattern associated with new entropy demands is possibly the cloud formations noted with the spreading of ice-crystal-containing tops of the cumulonimbus clouds seen in the flat anvil tops of such clouds (sometimes called thunderheads). The top of the cloud begins to flatten out, and cirrus-like clouds, consisting of ice crystals, spread out, creating a distinctive anvil shape. Because of the freezing temperatures high up in the atmosphere, cirrus clouds are usually made up of ice crystals, giving them a bright white appearance. These clouds form in flat sheets, which are not as thick as the cumulus and cumulonimbus (thundercloud) varieties. 

### 3.4. Analogy with Solidification Patterns (Microstructures)

The measurement of cloud parameters and relating them to the entropy generation rates presents several experimental difficulties because of the large scale. The entropy production rate can fluctuate spatially or temporally depending on the demand and scale—this is why a non-steady-state entropy rate production may show hysteresis for correlating with a particular morphology, recognized in both large and small-scale complex systems alike. It is thus valuable to compare diffuse interfaces at different scales with experimentally accessible analogs so that simulations of the weather can be carried out in laboratories.

Similar shape features observed in vertically developing clouds are seen in liquid-to-solid transformations [35,36,37,38,43,74]. There appear to be significant similarities between pattern section processes and the patterns themselves that are seen in controlled solidification and clouds (Figure 6). These pattern similarities in known condensed-phase transformations (laboratory-scale experimental systems) and the large-scale cells inside a cloud have never been identified before. The scale of protrusion or perturbation (see Figure 6 and Figure 7) inside a cloud is on the order of meters to tens of meters. The scale of dendrites and cells in phase transformations is typically a few to a hundred micrometers. However, the total amount of entropy generation per unit volume in a cloud (Equations (18)–(24)) and condensed-matter liquid–solid transformation to a cell or dendrite appears to be similar—as noted when comparing the numbers in Figure 2 and those reported in reference [17]. Morphological variations and entropy production rates in solidification are well-documented and correlated [10,13,18,19,22,43]. In condensed-matter solidification (crystallization), the analogy to multicellular clouds is the grains and dendrites and their boundaries as is noted when comparing the morphologies in references [41,42]. Such boundaries are regions of entropy concentrations that can influence the energy and entropy balance in a control volume [22]. The boundaries between multicellular clouds are not as well-characterized as those inside a crystalline solid. During liquid-to-solid transformation, finer cells, cell tips, and additional branching features called dendrites are seen in the evolution of liquid–solid morphology with conditions that promote diffuse interfaces and increased entropy generation rates [36,37,38]. The analogous nature of the breakdown of the front of a two-phase transformation in clouds and solidification appears similar (although quite different in scale). Figure 6 shows the similarities between cloud morphologies and solidification microstructures (with both a negative- and positive-interface temperature gradient) [36,74].

Equation (26) can be rearranged to give,

V_c_/SLR = 2ξ ^2^ [[ξ dΔ(*f_v_*(s_vpi_)/dt)] + [ρC_p_ln(T_avc_)V_c_]SLR/ΔT]/∆*ρ*_*k*_*f_v_*V_c_^2^ (27a)

Assuming that there is no precipitation, the bifurcation condition for vertical instability is thus given by:

V_c_/SLR = [(2ξ^2^ C_p_ln(T_avc_) ΔT/*f_v_*)]^0.5^/ΔT (27b)

Equation (27b) *has a very similar form* to the classic solidification bifurcation equations (Equation (27c)) for the onset of interfacial instability in solidifying binary alloys [35,36,37,38,43,74]. Equation (27d) can be compared with Equations (24) and (27a) for the similarities in entropy generation rates. In Equation (27c,d), V, G, D, and ΔT0 are the interface velocity, temperature gradient at the interface, solute diffusion constant, and solidification range, respectively [35,36]. SLI is the subscript for the solid–liquid interface region.

(V/G)_interface_ = (D/ΔT0) (27c)

[((dsgen/dt)T_si_T_li_)/G_sli_h_sl_] < (V/G_s_li) < 2[((dsgen/dt)T_si_T_li_)/G_sli_h_sl_] (27d)

The granular details in comparing Equation (27b,c) are left to future publications. The accurate form of the interface stability in Equation (27c) is given in reference [55], where the breakdown (bifurcation) condition is V^2^·f(T_av_, Co), where T_av_ is an average temperature in the two-phase region and Co is the average bulk chemical composition [13,36]. Note also that a wide diffuse-interface condition is noted (like the case of clouds) when the solidification occurs close to the T(C0) temperature, i.e., when the free-energy functions of the liquid and solid intersect in an alloy phase diagram. The T(C0) is the temperature and composition of equal free energy per mole for the phases. A circular repeating reaction with static steady-state patterns like the decaying Belousov–Zhabotinsky (BZ) reaction patterns at small scales is also known as a feature related to the entropy generation rate [69,70,71]. We have not yet identified any similar BZ reactions in clouds.

## 4. Summary, Comments, and Conclusions

There are several phenomena that a large-scale complex system like the troposphere can invoke to generate new entropy rates. These can span different scales of entropy production rates. The troposphere (a large-scale complex system) can choose high-velocity wind (with corresponding energy transfer to generate new entropy) in competition with or in addition to the entropy generation rate mechanism offered by cloud formation (which requires high water saturation in the atmosphere). The high-rate entropy generation clouds or rapid winds in the PBL (or higher regions) can respond competitively to demands on the entropy generation rate while attempting to maximize the entropy generation rate. The possible competition between winds and other entropy production methods by clouds and lightning are discussed below with comparisons with recent climate observations.

The low-complexity model presented in this article indicates that an increase in the Earth’s average surface temperature will trigger an increased entropy generation rate demand in the troposphere. The model indicates that the demand for the entropy generation rate is proportional to the second power of the surface temperature at the current rate of the Earth’s surface warming. A higher rate of entropy generation could lead to more intense weather from the chosen pathway selections that produce entropy. With a continued increase in the Earth’s surface and tropospheric temperatures, it is increasingly difficult to reach steady-state conditions. Although the model provides only a simple description of tropospheric phenomena and cloud behavior, it appears to validate the postulate of global warming leading to more severe weather.

The presence of specific types of clouds enables demand as well as increases the entropy generation rate requirements of the troposphere, depending on the net emissivity variations of clouds and dry Earth. Equilibrium horizontal clouds behave very differently from vertically developing clouds regarding the entropy generation rate. Entropy-generating instabilities in clouds lead to a higher updraft velocity, which means that cumulus and towering cumulus clouds will increase with surface warming (in the presence of higher water content in the atmosphere enabled by the warming temperature). A greater cloud cover, higher cloud tops, and denser clouds are associated with regions of more vigorous storms. The model in this article suggests that the average coverage and intensity of the Earth’s total cloud coverage will intensify and display more vertically growing clouds to respond to the demands for a high entropy generation rate. Although not rigorously discussed for clouds in the troposphere, it should be noted that a higher rate is significantly promoted by condensation following undercooling and rapid recalescence. It should be noted that there could also be a possibility of extremization of the first derivative of the entropy generation rate which could influence the speed of transition between two steady-state entropy generation rate conditions as well as enable metastable morphological variations in the cloud shapes. These metastable states could have similarities to the physics of what could be observed at critical points in a one-component phase diagram [28,29,30,53,54]. Almost all such states are relatively unknown or not adequately recorded for cloud morphologies.

Variations in cloud morphology and coverage can result in a rapid response to the entropy generation rate demand for a fixed location (and diurnal and seasonal time scales) of the Earth’s surface warming or as a response to the comparatively slower time scale of global warming. The short-term response of clouds can enable vertically developing cloud morphologies (see Stages 3 and 4 in Figure 7), which, coupled with lightning, can intensify rapidly, and only fizzle when the Earth below cools or when the condensed moisture that forms as a diffuse region (namely clouds) is discharged or dissipated. The long-term response of clouds to global warming is likely to include an increase in their coverage and the intensity of cloud discharges. Winds in the PBL commonly mitigated by topography may become less impeded. The frequency and strength of storms are related to such climatic factors as the average wind speed and direction, temperature, humidity, and sunlight, which are all impacted by the gradients in the entropy generation rate. As discussed in this article, the updraft velocity (controlled by buoyancy, heat-producing transformations, cold and warm front movements, and terrain) is critical to cloud pattern formation. However, in addition to the influence on the initial cloud-pattern formation, the updraft velocity may also impact the type of diffuse-interface phase partitioning of liquid and ice (like the transformation velocity, which impacts the partition function between solid and liquid in diffuse phase transformation [13,17,36,37]). Such derivative phenomena are possibilities that can influence the severity of future weather patterns in unexpected ways.

Several energy-based models show a recent intensification of heavy precipitation across almost all land regions of Eurasia and North America. The entropy generation model discussed in this article also indicates such an intensification over land masses is possible. Updraft velocities can skew otherwise accurate calculations and measurements of the average effective cloud coverage and, thus, the energy-balance calculations (for Earth surface warming). The tropospheric energy-balance calculations thus become problematic because the cloud emissivity changes for different cloud morphologies, which in turn impacts cloud heights (Equation (19)), cloud temperatures, updraft velocities (Equation (25)), and water pathways inside the clouds [39]. Cloud formation issues are a challenge to atmospheric energy-balance calculations [7,8,9,56,57,58,59,60,61,62,63,64,65] but could be more accessible to tackle with the entropy-balance model and calculations discussed in this article.

Low-pressure areas at mid-latitudes are regions of severe weather. Because there is considerably more evaporation as the surface temperature becomes warmer [3,4,5,6], the model presented in this article suggests the faster formation of clouds over land (compared to ocean clouds) in response to global warming. The Arctic region continues to experience the strongest warming trends—close to two to four times the global average [64,65]. When dry winds are actively blowing, the contribution of water vapor to the composition of the immediate atmosphere is low, thus favoring wind movement over cloud formation. In the Earth’s desert regions, ~30° north and south of the equator, the water-vapor concentrations are ~2.5–3% (by weight) on sweltering days. An upper limit of about 3.9% is found in tropical climates, where maximum rainfall is typically experienced. The least rainy place on Earth is near polar regions (not deserts). Evidence suggests that the wind speeds over the polar regions have increased with an uptick in global surface temperatures [89]. One explanation for the higher rate of surface warming at the poles may arise from the differences in entropy generation rates across mid-latitudes (where localized extremes are noted) and the polar regions that can lead to energy transfer to the polar regions [68,89]. When a low-pressure system is present in the lower troposphere, it may induce a high-pressure system in the upper layer of the troposphere [68]. Circulatory wind cells with rapid winds are triggered by the upper atmosphere's moisture content when it cannot find a mechanism with cloud formation to alter the weather, Thus, hotter winds could flow towards the polar regions from the mid-latitude regions. Compared to the equatorial troposphere, a possibility of a slower velocity of tropopause expansion over the Arctic regions could also increase the required entropy production rate per unit volume in the troposphere above the polar regions (Equation (9)). The warming rate indicates that the rate of entropy generation per unit volume over the poles can be remarkably high even though the surface temperature is lower at the poles than at the mid-latitudes, enabling the possibility of heightened spatial (primarily horizontal) energy fluxes (high wind velocity) in the regions surrounding the Arctic. Changes in the polar PBL are diurnal and rapid compared to the slow changes in the layer above the PBL. Temperature inversions are quickly enabled in the PBL, thus explaining nighttime rains—even while the Earth cools at night. Although any surface and tropospheric cooling lowers the demand for cloud-generated entropy in the troposphere, it may enable saturated atmospheric conditions to appear.

Other forms of entropy generation rate intensification phenomena can correspond with severe weather. For example, interactions between high-velocity wind and significant-water-containing clouds can lead to multicellular thunderstorms. Lightning can also transfer entropy out of a cloud, leading to a higher demand for entropy generation. The interaction of vertically developing thunderstorm clouds and high-altitude horizontal winds can cause rotating updraft clouds for relatively long periods. Several intense weather configurations have been illustrated by Abbot [87], reproduced in Figure 7 (see Stage 4). Such intense storms occur, sometimes accompanied by lightning when wind gusts interact with coastal clouds. Other possibilities of rapid entropy production include multicellular clouds and lightning (combined with high water and hail discharge rates) with wind rotation-enhanced entropy generators (Figure 7). Supercell updrafts and downdrafts remain separate as fast winds aloft carry raindrops, ice crystals, and hail out of the updraft.

Global warming is because of increased greenhouse gas emissions (GHGs). The GHGs trigger a demand for a higher entropy generation rate in the troposphere. This rate is enabled by changing cloud behavior or winds. Vertically developing cloud formations and other severe weather patterns are a method for rapidly responding to the increase in the demand for tropospheric entropy generation rate in the diurnal and global-warming time scales. If the GHG emissions are quickly reduced (for example, by eliminating fossil-fuel industrial heating by efficient electric heating methods), the requirement for increasing entropy generation rates will become low to zero (cannot be negative) and thus, delay the onset of ever-increasing intense and severe weather that the Earth is experiencing. Such changes will provide stability for the troposphere to recover to a steady-state condition.

## Figures and Tables

**Table 1:** Cloud types and their typical water content and vertical velocity development, adopted from reference [46]. Clouds with “cumulus” in the name show updraft velocities. Stratus clouds develop horizontally. Stratocumulus clouds are hybrids of layered stratus and multicellular cumulus clouds.

Cloud Type	Liquid Water Content (g/m^3^)	Measured Vertical Updraft/Velocity (m/s)	Volume Fraction of Water, *f_v_* in Clouds
cirrus	0.03	Small	3 × 10^−8^
fog	0.05	0.25	5 × 10^−8^
stratus	0.25–0.30	Small	(2.5–3) × 10^−7^
cumulus	0.25–0.30	1	(2.5–3) × 10^−7^
stratocumulus	0.45	0.5	4.5 × 10^−7^
cumulonimbus	1.0–3.0	10	(1–3) × 10^−6^

## Data Availability

No new data created or analyzed. Data sharing is not applicable.

## Appendix A. Description Scheme of Various Phenomena in the Key Equations for Tropospheric and Cloud Entropy Balance

**Relevant Equation**	**Expression**	**Type**	**Units**
Equation (5b)	(4A_Earth_Φε_χ_ σT_c_^3^/3)Δt	Entropy lost from the troposphere from radiation with the average cloud temperature	J/m^3^.K
Equation (5b)	(4σA_Earth_(1 − Φ)ε_α_ T_E_^3^/3)Δt	Entropy lost from the troposphere from the Earth’s surface that do not see cloud cover	J/m^3^.K
Equation (5b)	(4σT_sun_^3^/3)κ_1_ψΔt	Entropy lost from the troposphere from radiation from the Earth that is mitigated by clouds	J/m^3^.K
Equation (6)	hρC_p_(ln(T)) =	Entropy changes per unit area of the tropospheric slice	J/m^2^.K
Equation (6)	(4σψT_sun_^3^/3)Δt	Entropy changes per unit area of the tropospheric slice	J/m^2^.K
Equation (6)	(4σT_sun_^3^/3)κψΔt	Entropy exchange not captured in the other terms that arise from reflections	J/m^2^.K
Equation (6)	hd**s**_gen_/dtΔt	Entropy generation term in the troposphere	J/m^2^.K
Equation (6)	dΔδ/dt.Δt	Entropy exchange from evaporation and condensation that crosses the control volume boundary located at the Earth’s surface	J/m^2^.K
Equation (6)	ΛΔt	Tηε entropy-loss term associated with the catch-all electron jet (like lightning) that crosses the boundaries of the tropospheric control volume	J/m^2^.K
Equation (15c)	$SLR=-g(\frac{(1+(\Delta {S/R)r_{v}\varepsilon )}^{\;}}{(Cp+({\Delta S}^{2}{/R)r_{v}ɳ)}^{\;}})$	Near-saturation and saturation lapse rate	K/m
Equation (16)	2ε_χ_κ_1_T_c(av)_^3^/ε_α_.λT_E_^3^ > 1	Bifurcation condition	Dimensionless
Equation (17)	[ξ dΔ(*f_v_*(s_vpi_)/dt)]SLR/ΔT	Rate of entropy generation per unit volume with moisture exchange processes	J/m^3^.K.s
Equation (17)	[C_p_ln(**T_c(av)_**)V_c_]SLR/ΔT	Rate of entropy generation with tropospheric warming processes in the vertically expanding cloud	J/m^3^.K.s
